# The steppe species of gastrointestinal nematodes of small ruminants, with a focus on *Marshallagia*: climate as a key determinant

**DOI:** 10.1051/parasite/2011183261

**Published:** 2011-08-15

**Authors:** S. Meradi, B. Bentounsi, I. Zouyed, J. Cabaret

**Affiliations:** 1 Département des Sciences biologiques, Université de Batna Algérie; 2 Laboratoire de Parasitologie, Département des Sciences vétérinaires, Université Mentouri, Constantine Algérie; 3 INRA, Infectiologie animale et Santé publique 213 37380 Nouzilly France

**Keywords:** small ruminant, endoparasite nematode, *Marshallagia*, climate, steppe, host switch, petit ruminant, nématode endoparasite, *Marshallagia*, climat, steppe, capture

## Abstract

We intended to relate the geographic distribution of ruminant gastrointestinal nematodes in relation to steppe climate (and vegetation). Data are either from literature or from newly acquired/ available results. Simple or more sophisticated meteorological indices were used to characterize the climate. Regression analyses were used to correlate climatic factors and presence of endoparasites from steppe areas. The distribution of one (*Marshallagia*) out of five endoparasite genera was concentrated mostly in steppic areas whereas other species were found also in other areas. In wild hosts the distribution of *Marshallagia* was much larger from Sptizberg to New World (northern territories in Canada or extreme south of America). In domestic small ruminants the presence of *Marshallagia* was identified more frequently and constantly in the area of original domestication and its early diffusion (from Northern Africa to Kashmir, Caucasia). The distribution of this parasite was correlated to low rainfalls which were not the case for all other endoparasites. After host switch (reindeer or south America camelids), it has expanded in other climatic areas, either colder or dryer.

## Abstract (fr)

Notre objectif est de relier la distribution géographique des nématodes gastro-intestinaux des ruminants aux caractéristiques du climat (et de la végétation) steppique. Nous avons utilisé les données de la littérature et d’autres plus récentes que nous avons acquises. Nous avons recouru à des indices simples ou sophistiqués pour caractériser le climat. Des analyses de régression ont été utilisées pour estimer les relations entre les nématodes gastro-intestinaux et les facteurs climatiques. La distribution d’un genre (*Marshallagia*) sur les cinq rencontrés était surtout concentrée dans les zones steppiques, alors que les autres espèces étaient également rencontrées dans d’autres régions. Chez les hôtes sauvages, la distribution était beaucoup plus large, du Spitzberg au Nouveau Monde (territoires du nord du Canada et extrême sud de l’Amérique). Chez les petits ruminants domestiques, la présence de *Marshallagia* a été recensée de manière plus intense et plus constante dans les zones initiales de domestication ou dans ses diffusions proches (de l’Afrique du Nord au Cachemire ou au Caucase). La distribution de ce parasite était reliée à de faibles pluies, à l’inverse de ce qui était noté pour les autres nématodes. Ce n’est que lors de captures (renne, camélidés sud américains) que ce nématode a élargi son aire de distribution, dans des zones plus froides ou plus sèches.

## Introduction

It may look surprising that parasites would display macroecological and biogeographical patterns similar to those described for free-living organisms (Gueguan, 2006). In fact one may observe in many macroparasites similar ecological patterns as observed for terrestrial or marine free-living taxa (Poulin & Morand, 2004; Perez del Olmo *et al.*, 2009). Among the gastrointestinal parasite nematodes of herbivores, one part of the life is non-parasitic on the grass and they are clearly influenced by climatic environment (O’Connor *et al.*, 2006). The main difference is that their life within a host, who ingested infected herbage, is regulated to some extent by host susceptibility (Gaba *et al.*, 2006). One major difference in domesticated hosts is that they are often treated with anthelmintics, which may alter in the long run the diversity of macroparasites (Silvestre *et al.*, 2000). A second difference in these domesticated hosts is that they are transported from one site to another by their owner, sometimes on long distances for commercial reasons or availability of food. It could then be expected that ecological traits remains unexplainable due to these confounding effects of human management.

A previous study on the importance of climate and host species on occurrence of some macroparasitic nematodes (Ostertagiinae) of ungulates (mostly domestic ones and from 585 references altogether) did show that host species and climate played apparently a similar role (18% of occurrences explained equally either by range of host species or climates) (Suarez & Cabaret, 1991). Interspecific interactions in the gastrointestinal community are limited (Cabaret & Hoste, 1998) and this may simplify the understanding of climate/host factors. One of the genera of macroparasitic nematodes, *Marshallagia* sp., was associated with steppic climate and with domestic sheep and goats. However we know that it may be present in muskoxen or reindeer under polar climate (Halvorsen & Bye, 1999, in the Spitzberg) and in wild ungulates in several part of mountainous Europe (Italian Alps, among others: Zaffaroni *et al.*, 2000). The steppe climate comes under Köppen’s BS classification of climates (Viers & Vigneau, 1990). The B stands for dry climates, and the S for steppe climate. There is a huge difference between summer (up to 30 °C monthly temperature) and winter (sometimes below 0 °C monthly temperature) and the differences between day and night are also great. Some mediterranean climates (portuguese and hellenic) are not highly different from steppe climate but the number of dry-month is smaller. Steppe regions are often found in middle of continents and in the lee of mountains. Steppe vegetation may be found under different climates, and this renders the term steppe equivocal. Vegetation often consists in small xerophytic discontinuous grassland cover (opposed to prairie with continuous grass cover or savannah with tall grass). Steppe vegetation thus can be found in regions with very cold winter and hot summers (Central Asia, Eastern Europe) or under subarid mediterraneans climates (North Africa and several Middle-East countries). Thus steppe, either defined by climate or vegetation is not a very clear concept and we will substitute simple measures such as annual mean temperature or yearly rainfall in areas where *Marshallagia* is highly represented, since it was associated with steppe vegetation in a previous analysis (Suarez & Cabaret, 1991). *Nematodirus* species are also highly prevalent in steppe regions but they are also found in many different climatic areas (Morgan *et al.*, 2006). Genus such as *Haemonchus* (from temperate down to tropical areas where it predominates) or *Teladorsagia* (not found under the tropics but well adapted to temperate or cold climates) are found under a large range of climates; *Trichostrongylus* genera is nearly present under any type of climate. For example, North Africa has a large part of the inland under typical steppe climate and vegetation (Slimani *et al.*, 2010), and as expected, *Marshallagia* has been very frequently recorded (Morocco: Cabaret, 1984; Algeria: Bentounsi *et al.*, 2007 and Boulkaboul & Moulaye, 2006); but other trichostrongylid nematodes such as *Teladorsagia circumcincta*, *Haemonchus contortus*, *Trichostrongylus colubriformis*, *Trichostrongylus vitrinus*, *Trichostrongylus probolurus* and *Nematodirus filicollis* are also frequently recorded (Graber, 1979).

We intend to answer to the following questions: 1) is *Marshallagia* associated to particular climatic characteristics such as average temperature and annual rainfalls or rather to a set geographical locations in domestic sheep and goats? 2) is *Marshallagia* associated with other nematode species in sheep and goats? 3) could the wild hosts infection with *Marshallagia* help to decipher the distribution of this species?

## Materials and Methods

### Parasitological Methods

Parasitological examinations concern faeces or gastrointestinal tract of necropsied hosts. The differentiation of eggs in faeces is limited to *Marshallagia*, *Nematodirus* and other gastrointestinal nematodes (MAAF), 1986). The worms collected at necropsy are identified to species (Skrjabin *et al.*, 1954). The organs examined were abomasa (glandular stomach of ruminants) or abomasa and small intestines.

### Recorded Occurrences of *Marshallagia Marshalli* in Literature

**Fig. 1.**
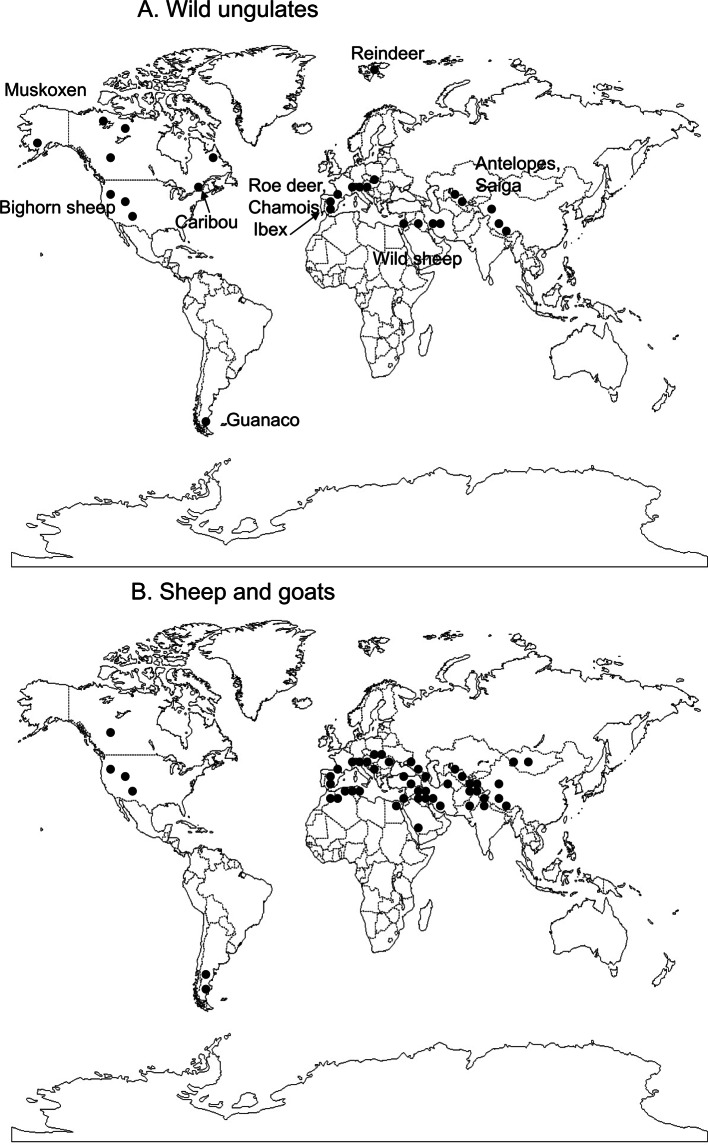
Geographic distribution of the nematode *Marshallagia marshalli* in wild ungulates (A) and domestic sheep and goats (B) established on records from 1930 to 2010.

The occurrences were obtained from older records (Skrjabin *et al.*, 1954) or more recent records using Web of Science – All data bases in May 2010. We also used parts of the records from Suarez & Cabaret, 1991, that were based on Helminthological Abstracts (1960-1989), Index Catalogue of Medical and Veterinary Zoology (1966-1982). It was a critical review of occurrences and the cattle occurrences were discarded since either Skrjabin *et al*. (1954) or ourselves in our unpublished investigations could not find *Marshallagia* in cattle. These occurrences are shown in Fig. 1A, B.

### Own Survey in Algeria

The studied farm (el Mader) is located in the North- East of Algeria (Department of Batna). The coldest month is January with 5.6 °C. The hottest month is July with 27.5 °C. Yearly rainfalls are 368 mm and the drought period extends from June to September. The climate is categorized as semi-arid with temperate winter or steppic. 30 weaned lambs were selected among the available lambs and were used as sentinels to monitor the infection. They were monthly examined for faecal egg counts and two to three lambs were necropsied monthly to identify the species of gastrointestinal nematodes (January 2008-March 2009).

### Meta-Analysis of Nematofauna in Steppe Areas

The sites were selected on the basis of constant presence of *Marshallagia* and detailed results that encompasses several seasons. The sites were also representative of a large geographic range with *Marshallagia*. Nine sites or periods were investigated regarding sheep faecal egg counts (Eastearn and western Algeria, Northwestern areas of China, Center area of Kazakhstan, and four areas of Northwestern Syria) with *Marshallagia* prevalence ranging from 10 (Kazakhstan) to 85% (our survey in Algeria) (Table I). Necropsies of hosts were performed to identify nematodes found in abomasa in nine sites or periods (Morocco, Mongolia, Kashmir, Syria, Spain) (Table II). The percentage of *Marshallagia* in the abomasum nematode community ranged from 3 (year three in Morocco) to 81 (Syria 4). Necropsies of hosts on all the digestive-tract were conducted in few sites (Algeria, Kazakhstan, Turkey-Van and Syria) and prevalences are shown (Table III).

**Table I.** Prevalence of nematodes groups in sheep and goats based on faecal egg counts.

	Site								
	Algeria (Batna)	Algeria	Algeria	China	Kazakhstan	syria 1	syria 2	syria 3	syria 4
						
	Eastern area: Present study	Western area: Tiaret 1	Western area: Tiaret 2	North- western areas	Center area: Chu	Northwestern areas: 1 to 4			
Reference		Boulkaboul *et al.*, 2006		Cai *et al.*, 2009	Morgan *et al.*, 2006	Giangaspero *et al.*, 1994			

Average yearly rainfalls and temperature	367 mm 17 °C	375 mm 16 °C		289 mm 11 °C	350 mm 8 °C	180 mm 15 °C			
Type of climate	Semi arid Steppe	Semi arid Steppe		Semi arid Steppe	Semi arid Steppe	Arid Steppe/Desert			

Gastrointestinal Nematodes* (%)	100	57	42	80	40	45	100	90	90
*Marshallagia*	85	28	21	67	10	23	25	38	37
*Nematodirus*	96	27	26	26	50	6	14	15	12

* other than *Marshallagia* and *Nematodirus*.

**Table II.** Proportions within the community of nematodes species of sheep and goats based on necropsies (abomasum).

	Site										
	Morocco			Mongolia		Kashmir	Syria 4	Spain			
	Moulay 1	Moulay 2	Moulay 3	Province 1	Province 2	Valley	North west areas	Leon 1	Leon 2	Leon 3	Leon 4
Reference	Cabaret, 1984			Sharkhuu. 2001		Tariq *et al.*, 2010	Giangaspero *et al.*, 1992	Diez-Banos. 1989			

Average yearly rainfalls	556			254		523	180	530	480	390	750
and temperature	18			0		13	15	12	12	11	11
Type of climate	Semi-arid/Sub-humid Steppe/Portuguese			Arid Steppe		Portuguese	Arid Steppe/ Desert	Portuguese	Portuguese	Steppe	Portuguese

Nematode species (%)											
*Marshallagia marshalli*	11	8	2	64	68	17	81	27	37	27	46
*Teladorsagia circumcincta*	32	50	44	69	77	30	92	97	97	96	100
*Trichostrongylus axet*	50	26	43	8	8	25	0	63	60	70	58
*Trichostrongylus vitrinus*	6	11	5	0	0	0	48	3	3	4	2
*Haemonchus contortus*	0	0	0	28	29	48	0	6	7	11	0

**Table III.** Prevalences of nematode species in sheep and goats: necropsies of all gastrointestinal-tract in highly *Marshallagia* infected sites.

	Site					Variance to mean ratio of prevalences
	Algeria (Batna)	Kazakhstan	Turkey (Van)	Syria 5	Syria 6
Reference	Present study	Morgan *et al.*, 2006	Cengiz *et al.*, 2009	Hörchner, 1964		

Average yearly rainfalls and temperature	367 mm 17° C	350 mm 8 °C	385 mm 9 °C	180 mm 15 °C		
Type of climate	Semi arid Steppe	Semi arid Steppe	Semi arid Steppe	Arid Steppe/Desert		

Nematode species (%)						
*Marshallagia marshalli*	100	93	85	50	90	4.6
*Teladorsagia circumcincta*	100	20	75	18	34	26.8
*Haemonchus contortus*	67	30	40	5	3	24.3
*Trichostrongylus axei*	17	60	33	1	2	26.9
*Trichostrongylus vitrinus*	92	0	0	14	28	54.6
*Trichostrongylus colubriformis*	58	55	0	8	10	29.8
*Trichostrongylus probolurus*	3	10	19	2	6	5.9
*Nematodirus helvetianus*	100	0	0	0	0	*Nematodirus* all species 31.6
*Nematodirus filicollis*	84	15	0	14	14	
*Nematodirus spathiger*	84	35	65	6	6	
*Nematodirus oiritianus*	0	0	75	0	0	

### Climatological Parameters and Analysis of Data

The rainfalls (R) are below 40 cm per year in steppe climate. The rainfalls in steppe climates are related to yearly average temperatures (t in °C) in the following ways: R in cm < 2 t when dry season occurs in summer, R < (t + 14) when dry season is in winter and R < 2 (t + 7) when no season effect is observed. We used the difference between R - 2 t, R - (t + 14) or R - (2 t + 14) as an index of departure from steppe climate either in dryness (negative values) or excess of humidity (positive values) and we call it DS (departure from steppe). The lowest value was in, Syria (- 12) and the highest in León 4 (53). The De Martonne’s aridity index I is calculated as follows: I = R in mm / (t + 10) and is between 5 to 10 for dry steppe. It ranged from 7.2 (Syria) to 35.7 (León 4).

All the statistical analyses were performed using SPSS 11.5 (regressions) or MVSP 3.1. (cluster analyses based on UPGMA and Spearman rank correlations) softwares.

## Results

### Geographic Distribution of *Marshallagia* in Wild and Domestic Ungulates

The distribution of *Marshallagia* in wild ungulates (Fig. 1A) was relatively extended among continents but restricted to few sites. Most of the distribution was associated to mountains except in reindeer from Spitzberg. The wild ungulates disseminated *Marshallagia* into polar or boreal climates. The infected domestic sheep and goats (Fig. 1B) were also recorded in mountain regions either in the New World (Rocky mountains and Appalachians in North America and the Andes in South America) or in the Old World (Alps, Atlas, Taurus, Caucasus, Himalaya, and Altai). The majority of occurrences were located in Eurasia (45 out of 51 sites) of which half were in the original areas of domestication (Middle-East and Northern India).

### Nematode Faunistic Similarity Between Sites and Periods

**Fig. 2.**
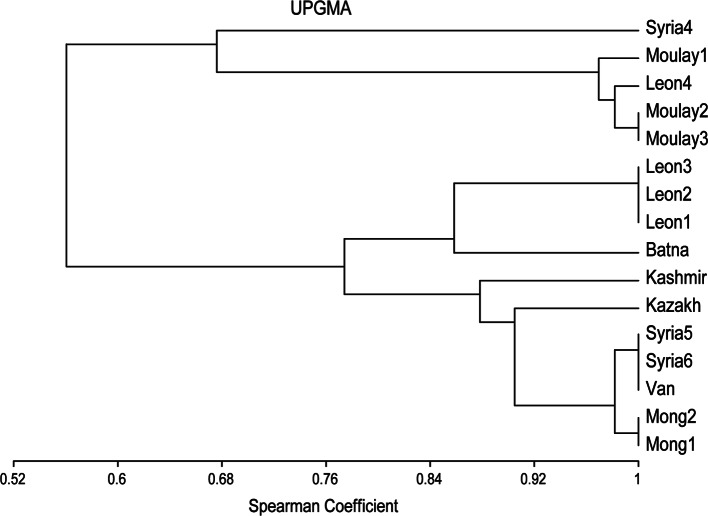
Prevalence of nematode species located in abomasum: data of 16 surveys in steppe and cluster analysis using UPGMA and Spearman correlation coefficient.

The faecal egg counts provide a first gross evaluation on the nematode fauna (Table I). The prevalence of *Marshallagia* ranged from 10 (Kazakhstan) to 85% (Algeria). It should be noted that it may vary much from different sites in the same country (21 to 81% in Algeria). The importance of other nematodes characterized different sites with the high proportion of *H. contortus* in Kashmir valley and in Mongolia, of *T. vitrinus* in Syria 4, of *Trichostrongylus axei* in Morocco and León (Table II). When considering the total fauna and prevalence of species in sites with high prevalence of *Marshallagia* (> 50% hosts), most of the other species were highly variable among sites (Table III). The ratios variance to mean of prevalences were low for *Marshallagia* and *T. probolurus* and were high for all the other species or for *Nematodirus* genus. On the UPGMA performed on the prevalences of parasites in abomasa (Fig. 2) an Eurasian group appears (Syria, Turkey, Kazakhstan, Kashmir and Mongolia). Two groups are climatically driven: one group is in relation to the subhumid climate (Morocco and León 4 in Spain) and another group corresponds to arid climate (León 1 to 3 in Spain and Batna in Algeria).

### Relationship Between Climate and Presence of *Marshallagia* and Other Nematode Species

**Fig. 3.**
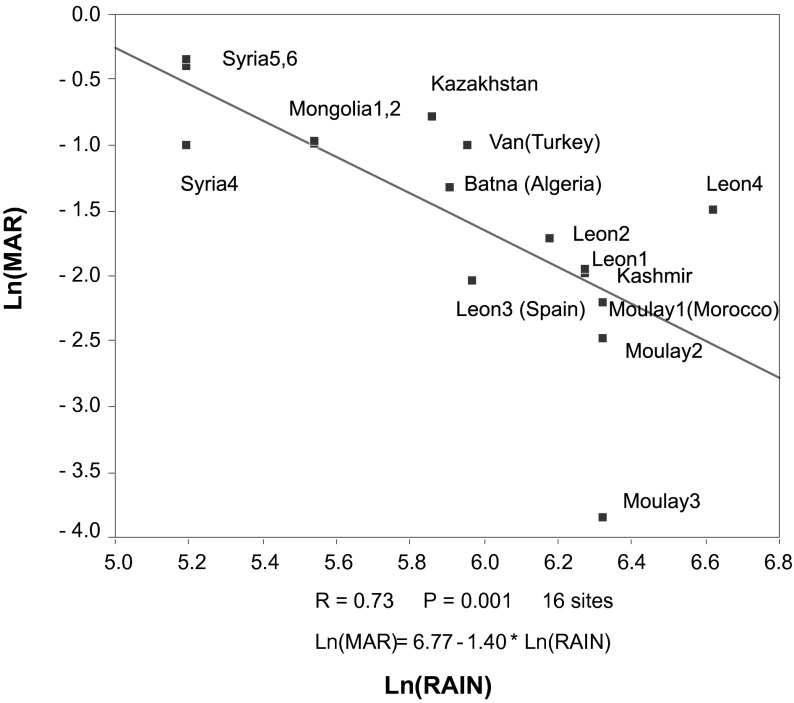
Linear relationship between prevalence of the nematode *Marshallagia marshalli* and rainfall in 16 surveys in steppe. (Ln: neperian logarithm, R: regression coefficient and P probability of type 1 error).

The significant relations between prevalence and climate indicators were: *Marshallagia* with rainfall (rs = - 0.78; n = 16), Martonne index (rs = - 0.54) and DS (rs = - 0.60), *T. circumcincta* (Martonne index rs = 0.72, DS rs = 0.79; n = 16), *T. axei* (rainfall rs = 0.77, DS rs = 0.53; n = 16), and *Nematodirus* (rainfall rs = 0.73; n = 9). Rainfall was the best indicator for prevalence of *Marshallagia* and other nematode species. A significant linear regression was established between the prevalence of *Marshallagia* and rainfall (Fig. 3). Contrasting to most other nematode species, lower rainfall was associated to higher *Marshallagia* prevalence of infection.

## Discussion

Maximum co-speciation has been considered as the norm, but it must be replaced by a more general model of evolution that includes host switching to understand the geographic distribution of host parasite systems. Host-switching and geographical dispersal of parasites are common phenomena in natural host-parasite systems (Hoberg & Brooks, 2008). These events of high importance at an evolutionary scale of time may also play a role at the historical scale particularly for domestic hosts and their parasites. The geographic range of domestic herbivores has expanded enormously, as well as the number of hosts (of the original host species and also other species the parasite was in contact with). It remains however difficult to decipher the role of physical environment (climate included) and geographic location per se in the presence of domestic animals in a region. The goat may well have been domesticated a little earlier than other domestic ruminants. The ancestral wild stock exists, is the wild goat (*Capra aegagrus*), which is found from Anatolia to Pakistan, in the Neolithics (Moutou & Pastoret, 2010). It is probable that there were two invasions to Africa, in the 5th and in the 3rd millennium BP. Sheep were introduced to the southern Levant as fully domesticated by the end of the 9th millennium BP (Haber & Dayan, 2004) and presented similar invasions. Thus the area extending from North Africa to Pakistan and possibly Caucasus (supported by the genetic analysis of wild and domesticated sheep: Hienleder *et al.*, 2002) constitutes the original area of domestic sheep and goats at the end of the Neolithic. The presence of the parasitic nematode *Marshallagia* corresponds in part to the origin of the small ruminants although it has expanded to Spain and mountain areas of several parts of Europe. It has also clearly expanded geographically through the dissemination of domestic sheep and goats. This parasite has been found in wild *Ovis* (prevalence 11.5% similar to domestic sheep, in the data set of Suarez & Cabaret, 1999) or *Capra* (prevalence 15.5% in the same data set) but was captured by reindeer, muskoxen, and several species of cervids, among which Caribou. There is thus a contradiction to the relatively limited area of *Marshallagia* in sheep and goats and its capabilities to invade a wide variety of hosts and climates (Fig. 1A). It is an illustration of the Beijerinck’s law “Everything is everywhere, but the environment selects” (cited in Perez del Olmo *et al.*, 2009): “everywhere” concerns mostly wild ungulates and small ruminants and “environment selects” is particularly true for the nematode infection of worldwide distributed domestic sheep and goats.

Climate is clearly a key for the distribution of gastrointestinal nematodes of sheep and goats (O’Connor *et al.*, 2006). Moreover, sheep and goats are present throughout the world and if distribution of parasitic nematodes was only host driven, they should be found everywhere, which is not the situation and particularly for *Marshallagia*. Rainfalls play a major role and temperature (at least in steppe areas) is less important. More sophisticated climatic indices (Martonne or index of departure from steppe climate) are not more efficient for prediction of nematode prevalence in a site. The relation to rainfalls was negative for *Marshallagia* and was positive for all other species. In fact the highest prevalence was observed in areas with low rainfall (180 to 385 mm per year). The seasonal dynamics indicates that October-November in Morocco is the period with higher intensities (Cabaret, 1984); a similar trend was noted in Algeria (our unpublished results), in Spain (Diez-Baños, 1989) as well as in Uzbekistan (Oripov, 1982). This means that infection should occur at the end of the dry-season or the very beginning of rainy season. This seasonality corroborates the climatic environment needed for *Marshallagia*, a relative dryness. The capability to withstand dryness is however limited: there is no development from May to September of *Marshallagia* in very arid steppe of Uzbekistan (Marchanov *et al.*, 1985). The parasite community, with the exception of *Marshallagia* was highly variable (variance to mean ratio was high for all other specie that were largely represented) and there was no community that could be identified for steppic climate. This climate is then characterised by a single genera (*Marshallagia*) which is represented by very few species. In the absence of molecular genetic studies it is yet difficult to understand if there was an expansion with a relatively generalist species of parasite (able to survive in different hosts) or if there was a local genetic differentiation between parasitic isolates.
